# Deep Learning-Assisted Accuracy Improvement in Bladder Cancer Staging of Spectrum-Aided Visual Enhanced Cystoscopy Images

**DOI:** 10.3390/bios16080445

**Published:** 2026-08-16

**Authors:** Kuan-Hsun Huang, Yu-You Liu, Chia-Chien Wu, Chia-Ling Chen, Jie-Lun Hsieh, Lung-Hsiang Chuo, Hsiang-Chen Wang

**Affiliations:** 1 Department of Urology, Dalin Tzu Chi Hospital, Buddhist Tzu Chi Medical Foundation, No. 2, Minsheng Road, Dalin, Chiayi 62247, Taiwan; lqcat921@gmail.com; 2 Graduate Institute of Opto-Mechatronics, Department of Mechanical Engineering, National Chung Cheng University, 168, University Rd., Min Hsiung, Chiayi 62102, Taiwan; wow60659@gmail.com (Y.-Y.L.); nosehair91@gmail.com (C.-C.W.); jolincchen@gmail.com (C.-L.C.); jarebox1917@gmail.com (J.-L.H.); 3 Department of Surgery, Urological Surgery Division, Kaohsiung Armed Forces General Hospital, 2, Zhongzheng 1st. Rd., Lingya District, Kaohsiung City 80284, Taiwan

**Keywords:** bladder cancer, hyperspectral imaging, Spectrum-Aided Visual Enhancer (SAVE), computational optical biosensing, deep learning, YOLOv8, artificial intelligence

## Abstract

Recent statistics reported by the World Health Organization and the International Agency for Research on Cancer indicate that the global incidence of bladder cancer has continued to increase in recent years, particularly in industrialized countries. Therefore, the timely diagnosis of early-stage bladder cancer is of great clinical importance for improving patient prognosis and treatment outcomes. In this context, computational optical sensing frameworks that integrate Spectrum-Aided Visual Enhancer (SAVE) technology with cystoscopy have attracted significant attention to overcome the limitations of conventional visual data interpretation. In this study, an AI-driven optical biosensing framework was evaluated using 1372 white-light cystoscopy (WLC) images of bladder cancer (RGB-WLC) collected in collaboration with Chung Shan Medical University Hospital. Hyperspectral conversion technology was applied to extract precise spectral information from the white-light images. Subsequently, dimensionality reduction was performed based on the characteristic wavelengths of narrow-band imaging cystoscopy at 415 nm and 540 nm to generate hyperspectral reconstructed narrow-band images. The images were categorized into Ta stage (Ta), above T1 stage (Above T1), and four additional classes. The dataset was divided into training and testing sets to establish both a standard white-light cystoscopy model (RGB-WLC) and an advanced hyperspectral biosensing model utilizing the YOLOv8 architecture for enhanced pattern recognition. Model performance was evaluated using sensitivity, *F*1-*score*, and overall accuracy. The standard RGB-WLC model achieved an accuracy of 0.852, whereas the SAVE-based biosensing model achieved an accuracy of 0.948, representing an improvement of approximately 11.27%. The results demonstrate that combining algorithmic hyperspectral reconstruction with deep learning architectures effectively addresses the challenges of clinical data interpretation and significantly enhances the detection and staging performance of bladder cancer imaging.

## 1. Introduction

The bladder is an essential component of the human urinary system and is primarily composed of the bladder wall and the epithelial cells lining its inner surface. The bladder wall consists of the epithelial layer, submucosa, detrusor muscle layer, and outer membrane layer. Among these structures, urothelial cells play an important role in preventing harmful substances in urine from penetrating the underlying tissues. Bladder cancer remains one of the most common malignant tumors of the urinary system, with its global incidence continuously rising in recent years [1,2,3]. Due to its high recurrence rate and the frequent need for long-term endoscopic surveillance, it poses a severe clinical and economic burden on healthcare systems worldwide [4,5,6]. Consequently, addressing the diagnostic challenges associated with early-stage lesions is critical to reducing mortality and improving long-term patient outcomes [7,8,9].

**Figure 1:**
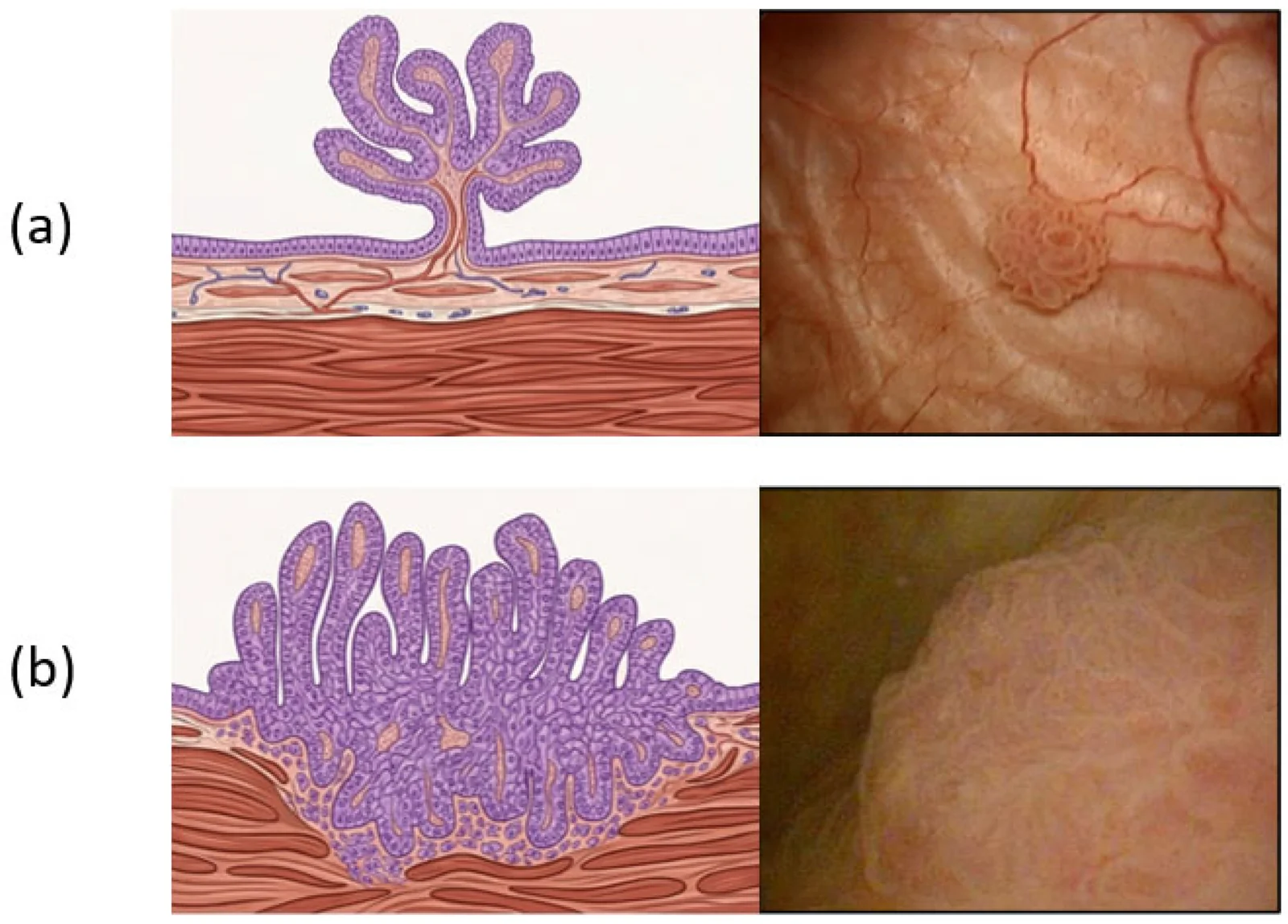
Morphological Types and Representative Images of Bladder Tumors. (**a**) Non-muscle-invasive bladder tumor (representative image of Ta stage) (**b**) Muscle-invasive bladder tumor (representative image of T2 stage, categorized under the Above T1 stage class).

Bladder cancer is mainly classified into two categories: non-muscle-invasive bladder cancer (NMIBC) and muscle-invasive bladder cancer (MIBC) [10,11,12,13]. Non-muscle-invasive bladder cancer (NMIBC) encompasses Ta stage, carcinoma in situ (CIS), and T1 stage [14,15,16,17]. Specifically, Ta stage tumors are non-invasive papillary carcinomas confined to the mucosal epithelium, as illustrated in Figure 1a. Carcinoma in situ (CIS) represents a flat, high-grade non-invasive lesion confined to the urothelium and is regarded as an early manifestation of aggressive bladder cancer. Progression to T1 stage signifies a more advanced NMIBC stage where cancer cells have invaded the submucosal layer (lamina propria) but have not yet penetrated the detrusor muscle layer [18,19,20]. In addition, carcinoma in situ (CIS) is considered a high-grade precancerous lesion and is generally regarded as an early stage of bladder cancer [21,22].

In contrast, muscle-invasive bladder cancer (MIBC), as shown in Figure 1b, is characterized by tumor invasion into the bladder muscle layer. These lesions are often more irregular and exhibit poorly defined boundaries, resulting in a poorer prognosis due to the increased likelihood of cancer cell dissemination into surrounding tissues or adjacent organs [23].

According to the 8th edition of the tumor–node–metastasis (TNM) staging system for bladder cancer published by the American Joint Committee on Cancer (AJCC) [24], TNM classification consists of T (Tumor), which describes the primary tumor size and depth of invasion; N (Node), which indicates the extent of regional lymph node involvement; and M (Metastasis), which represents the presence or absence of distant metastasis. Appropriate definitions are assigned based on the pathological characteristics and degree of invasion according to the Tumor–Node–Metastasis (TNM) subclassifications. In this study, the AJCC grading criteria were adopted to classify bladder cancer into Ta stage and above T1 stage categories [25].

In recent years, significant advancements have been achieved in bladder cancer detection technologies, particularly in cystoscopic imaging, which has improved diagnostic accuracy and enabled earlier detection of lesions. Common diagnostic methods include white-light cystoscopy (WLC) [26], narrow-band imaging (NBI) [27,28,29], blue-light cystoscopy (BLC) [30], and urine cytology examinations [31,32,33], which are increasingly supported by artificial intelligence algorithms for enhanced diagnostic accuracy [34,35,36]. These techniques assist physicians in identifying the location and extent of tumors. However, early-stage bladder cancer lesions often exhibit low contrast and indistinct boundaries, making them difficult to detect using conventional imaging modalities. In addition, traditional white-light cystoscopy (WLC) still has limitations in detecting flat lesions, such as carcinoma in situ (CIS), which may lead to missed diagnoses or underestimation of tumor extent [37,38,39].

Although narrow-band imaging (NBI) and blue-light cystoscopy (BLC) technologies can enhance lesion visualization, diagnostic consistency may still be affected by operator experience. Therefore, improving the visualization capability of early-stage lesions and establishing more objective interpretation standards remain important challenges in bladder cancer diagnosis. To overcome these limitations, advanced optical biosensing approaches, such as hyperspectral imaging (HSI) technology, have been introduced into medical image analysis [40,41,42,43,44,45,46]. This technique is capable of acquiring continuous spectral information within the visible light spectrum (approximately 380–780 nm), enabling different tissue types to exhibit distinct spectral responses at specific wavelengths, thereby significantly improving sensitive and selective lesion identification. Previous studies have demonstrated that significant spectral differences exist among bladder tissues at specific wavelengths, particularly at 415 nm and 540 nm, which are strongly associated with vascular and mucosal structural characteristics [27,28,29]. These spectral features can further enhance the contrast between lesion regions and surrounding normal tissues.

Based on this concept, the present study introduces Spectrum-Aided Visual Enhancer (SAVE) technology to transform conventional white-light cystoscopic images into simulated narrow-band images containing high-dimensional spectral data. The conversion process establishes a transformation model based on the correspondence between image features and spectral data to reconstruct the spectral characteristics of the original images, thereby enhancing lesion detection capability. The detailed conversion procedures and algorithms are described in the Supplementary Materials. Compared with conventional imaging modalities, hyperspectral biosensing can simultaneously provide both spatial and spectral information, allowing different tissues to exhibit unique optical characteristics and thereby improving the differentiation between tumor and normal tissues [47,48]. Previous studies have also demonstrated that this technology has been successfully applied to various tumor detection tasks, highlighting its promising clinical application potential [49,50,51,52,53,54]. While hardware implementations of such optical sensors have matured, the primary bottleneck in practical applications often lies in the automated processing and accurate interpretation of high-dimensional data. Modern artificial intelligence (AI) techniques offer unprecedented capabilities to address these challenges [55,56]. Among these approaches, the YOLO series models possess the advantages of real-time object detection and high accuracy [57,58,59], making them uniquely suited to provide unmatched pattern recognition for complex endoscopic biosensing data. Therefore, this study integrates algorithmic hyperspectral reconstruction technology with modern AI processing techniques to extract spectral features associated with different stages of bladder cancer and employs the YOLOv8 model for advanced image recognition and classification [60]. This synergistic framework aims to evaluate the potential of AI-enhanced optical biosensing in expanding the current boundaries of early bladder cancer detection and staging.

## 2. Materials and Methods

### 2.1. Dataset Collection and Image Acquisition

**Figure 2:**
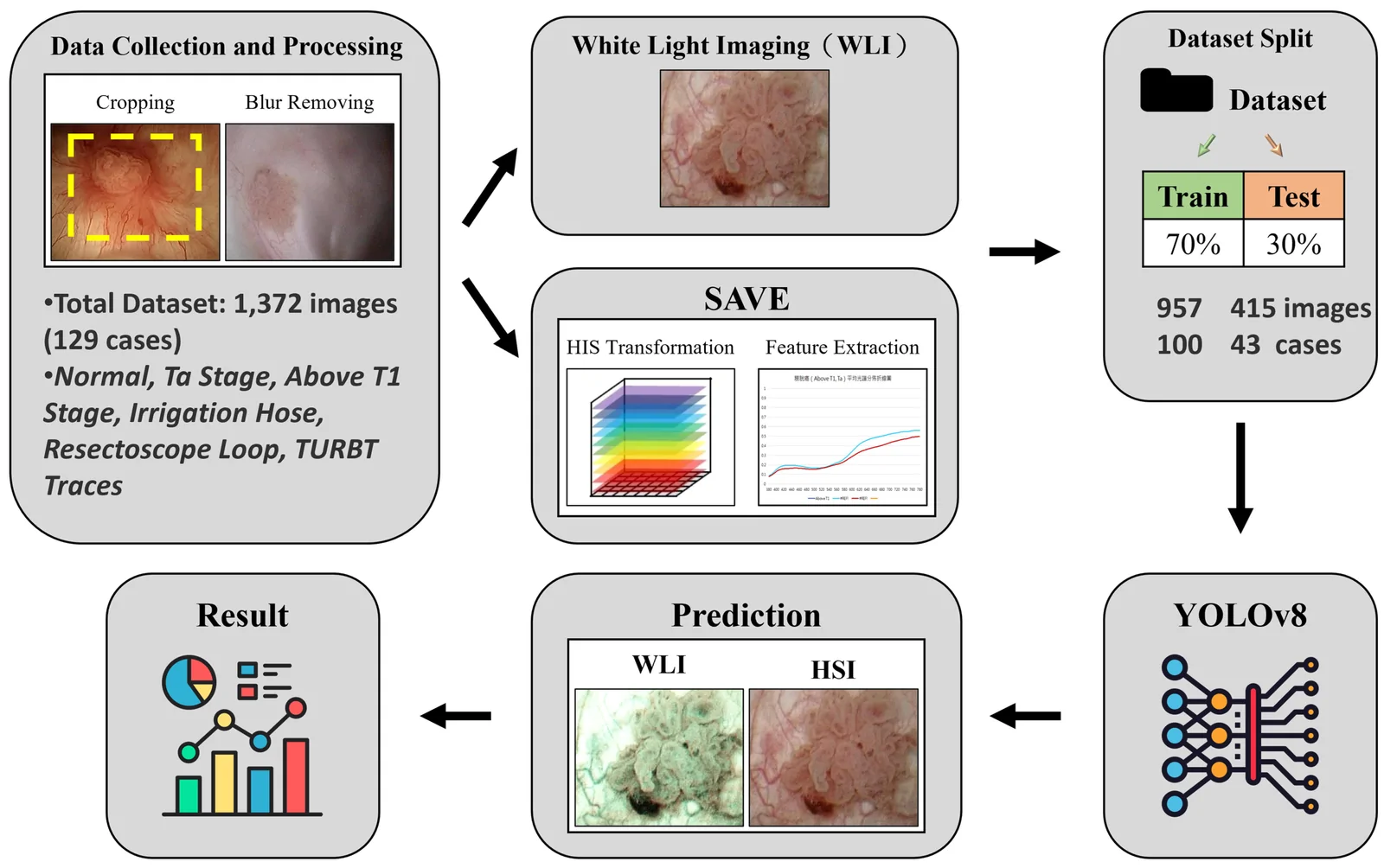
Schematic Diagram of the Experimental Workflow. The framework includes cystoscopic image collection (1372 images across 129 clinical cases), preprocessing, white-light cystoscopy (WLC) and Spectrum-Aided Visual Enhancer (SAVE) hyperspectral reconstruction, dataset partition into training (70%, *n* = 957 images from 100 cases) and independent testing sets (30%, *n* = 415 images from 43 cases), YOLOv8 model training, and diagnostic prediction across tissue categories (Normal, Ta, Above T1) and non-tissue surgical equipment artifacts (Irrigation Hose, Resectoscope Loop, TURBT).

The experimental workflow of this study is illustrated in Figure 2. Clinical cystoscopic images were collected by Dalin Tzu Chi Hospital, Kaohsiung Armed Forces General Hospital, and acquired using a white-light cystoscope (Stryker 4.0 mm 12° IR Filtered Autoclavable Cystoscope; Stryker Endoscopy, San Jose, CA, USA).

All images were individually extracted and cropped to obtain regions of interest. Blurred, low-quality, and clinically unsuitable images were excluded during the preprocessing stage. A total of 129 clinical cases were included in this study, yielding 1372 high-quality white-light cystoscopic images after image screening. According to the bladder cancer classification criteria established by the American Joint Committee on Cancer (AJCC) and the TNM staging system, tissue images were categorized into three clinical diagnostic categories: Normal bladder tissue (Normal), Ta stage tumors (Ta), and T1 stage or above tumors (Above T1). To further clarify the histological and morphological subtypes, the Ta stage includes papillary Ta lesions and flat lesions/carcinoma in situ (CIS), while the Above T1 stage encompasses T1 submucosal invasive tumors and T2 or higher muscle-invasive bladder cancers. Furthermore, three non-tissue categories representing surgical instruments and artifact traces were defined: Irrigation Hose (referring to fluid irrigation tubing, formerly “Hose”), Resectoscope Loop (referring to electrocautery resectoscope loop instruments, formerly “Loop”), and transurethral resection of bladder tumor traces (TURBT).

To evaluate model performance, the total 1372 images (129 cases) were split into a training set (70%, *n* = 957 images) and an independent testing set (30%, *n* = 415 images) as shown in Table 1. The training set consists of Normal tissue (50 cases, *n* = 116), Ta stage (16 cases, *n* = 146; papillary Ta: 8 cases/72 images, flat Ta/CIS: 8 cases/74 images), Above T1 stage (34 cases, *n* = 124; T1: 20 cases/74 images, T2+: 14 cases/50 images), Irrigation Hose (*n* = 102), Resectoscope Loop (*n* = 168), and TURBT (*n* = 301). The testing set consists of Normal tissue ([20] cases, *n* = 50), Ta stage (7 cases, *n* = 65), Above T1 stage (16 cases, *n* = 55), Irrigation Hose (*n* = 44), Resectoscope Loop (*n* = 72), and TURBT (*n* = 129). The clinical feasibility and baseline performance of this specific dataset (1372 images across 129 cases) was initially explored in our recently published work, which evaluated standard CNN backbones and a YOLOv5 model under an 80:20 patient-wise split [61]. To build upon those findings and establish a more rigorous evaluation standard, the current study implements a more stringent 7:3 independent split (strictly monitored at the patient-case level to prevent any spatial or temporal data leakage) and systematically benchmarks the next-generation YOLOv8 network architectures.

### 2.2. Hyperspectral Reconstruction and SAVE Image Generation

**Figure 3:**
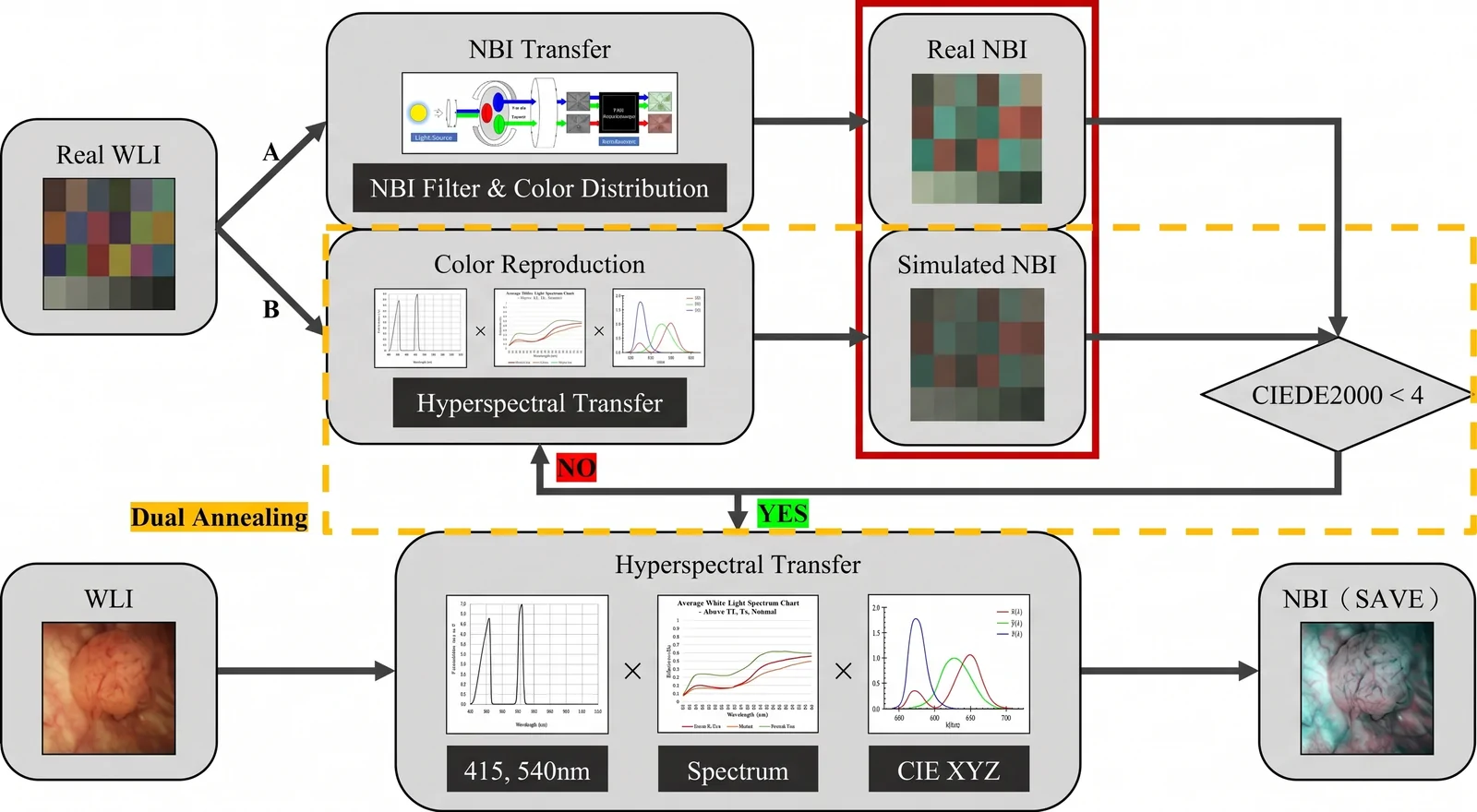
Workflow of Hyperspectral Conversion for Simulated Narrow-Band Image Generation.

Figure 3 illustrates the workflow of the Spectrum-Aided Visual Enhancer (SAVE) algorithm for converting conventional white-light cystoscopy (WLC) into hyperspectral reconstructed narrow-band images. In this procedure, white-light images of a 24-color checker were transformed through narrow-band filtering and color distribution conversion to generate corresponding narrow-band images. To ensure spectral consistency, color reproduction was performed using the white-light images of the 24-color checker. The Dual Annealing algorithm was applied to determine the optimal spectral distribution by minimizing the color difference between the simulated narrow-band images and the reference narrow-band images. The generated spectra were evaluated using the CIEDE2000 color difference metric. When the simulated spectra satisfied the predefined color difference criteria, the optimized narrow-band light source spectrum, hyperspectral reflectance spectra, and CIE XYZ color matching functions were integrated to reconstruct the final SAVE images.

In this procedure, white-light images of an X-Rite 24-color checker were acquired using the clinical cystoscope and transformed to generate corresponding narrow-band representations. To mathematically validate the reconstruction fidelity against physical ground truth, benchtop reflectance spectra of all 24 targets were acquired using a high-resolution spectrophotometer ( QE65000, 380–780 nm, Ocean Optics, Inc., Orlando, FL, USA). Principal component analysis retained 99.99% of the cumulative spectral variance within 12 principal components. A Dual Annealing optimization algorithm was applied to derive the transformation matrix by minimizing color discrepancies under the CIEDE2000 metric. Following calibration, the mean root-mean-square error (RMSE) between measured and reconstructed spectra across all 24 color patches was 0.058 for white-light conditions and 0.096 for narrow-band simulation, with mean CIEDE2000 color differences (ΔE_00_) reduced to 2.99 and 3.95, respectively (Supplementary Tables S1–S3 and Figures S7 and S8). The optimized transformation model was subsequently integrated with the characteristic absorption peaks of hemoglobin at 415 nm and 540 nm to generate the final SAVE images, highlighting tissue microvascular structures.

### 2.3. Deep Learning Framework

To evaluate the effectiveness of the Spectrum-Aided Visual Enhancer (SAVE) approach for bladder cancer recognition, two independent image classification models were developed using conventional white-light cystoscopy (WLC) images and SAVE images, respectively. This study employed the YOLOv8 classification framework (YOLOv8-cls) developed by Ultralytics. The YOLOv8x-cls model was adopted as the primary classification architecture. Both the WLC and SAVE models were trained using identical network architectures and training strategies to ensure a fair comparison between the two imaging modalities.

A total of 1372 images across 129 clinical cases were included in this study. To evaluate model performance while preventing data leakage, the dataset was partitioned at the patient-case level into a 70% training set (*n* = 957 images from 100 cases) and a 30% hold-out evaluation set (*n* = 415 images from 43 cases). Images from any given patient were assigned exclusively to a single split. Standard default YOLOv8x-cls hyperparameters and transfer learning settings were applied without iterative hyperparameter search on the hold-out set. The hold-out dataset served strictly to monitor epoch-wise loss convergence—terminating training at epoch 207 for the SAVE model and epoch 224 for the WLI model upon loss stabilization—ensuring an unbiased assessment of classification metrics. Following model training, performance evaluation was conducted using the testing dataset. Classification performance was assessed using sensitivity, precision, accuracy, and *F*1-*score*. For robustness analysis, three YOLOv8 model scales, including YOLOv8n-cls (Nano), YOLOv8s-cls (Small), and YOLOv8x-cls (Extra Large), were further evaluated to investigate the influence of model complexity on classification performance.

To prevent model overfitting and ensure robust generalization, several regularization strategies were implemented. First, an independent testing set (30%, *n* = 415) was strictly held out and never exposed during model training or hyperparameter optimization. Second, standard online data augmentations (including random rotations, flips, and spatial scaling) were applied to prevent feature memorization. Third, training convergence was continuously monitored with early stopping mechanisms, terminating training at epochs 207 (SAVE) and 224 (WLC) upon loss stabilization. Finally, ablation studies across various model scales (YOLOv8n to YOLOv8x) were conducted to verify that classification gains stemmed from spectral feature enhancement rather than over-parameterization.

### 2.4. Performance Evaluation Metrics

Model performance was evaluated using sensitivity, precision, accuracy, and *F*1-*score*. Sensitivity was selected as the primary evaluation metric because accurate detection of bladder cancer lesions is clinically important.

Sensitivity, also referred to as recall or true positive rate (TPR), measures the proportion of actual positive samples that are correctly identified by the model, as defined in Equation (1):

(1) $$Sensitivity=\frac{TP}{TP+FN}$$

Precision measures the proportion of predicted positive samples that are correctly classified, as shown in Equation (2):

(2) $$Precision=\frac{TP}{\left(TP+FP\right)}$$

Accuracy represents the overall proportion of correctly classified samples among all evaluated samples, which is calculated using Equation (3):

(3) $$Accuracy=\frac{\left(TP+TN\right)}{\left(TP+TN+FP+FN\right)}$$

The *F*1-*score* is the harmonic mean of sensitivity and precision and provides a balanced evaluation of classification performance, according to Equation (4):

(4) $$F1\text{-}score=\frac{2\;\times \;Sensitivity\times Precision}{Sensitivity+Precision}$$

where *TP*, *TN*, *FP*, and *FN* denote true positives, true negatives, false positives, and false negatives, respectively. Higher values of these metrics indicate better classification performance.

### 2.5. Statistical Analysis

Statistical analyses were performed using Python (v3.10; SciPy v1.11.2 and Statsmodels v0.14.0) to evaluate and compare the diagnostic performance between the conventional white-light cystoscopy (RGB-WLC) model and the Spectrum-Aided Visual Enhancer (SAVE) model on the independent testing dataset (*N* = 415). Because both models were evaluated on the identical paired image set, paired statistical testing was applied. Specifically, McNemar’s test for paired nominal data was performed on 2 × 2 matched contingency tables to determine whether differences in overall classification accuracy and category-specific sensitivities (e.g., Ta stage and Above T1 stage) were statistically significant. Two-sided *p*-values < 0.05 were considered statistically significant. Point estimates of overall accuracy, sensitivity, precision, and *F*1-*score* are reported alongside their binomial 95% confidence intervals (95% CIs) calculated via the Wilson score method with continuity correction. Confusion matrix analysis was conducted to examine category-specific error distributions. In addition, robustness and ablation analyses across different model scales (YOLOv8n-cls, YOLOv8s-cls, YOLOv8x-cls) and convolutional backbones (ResNet-50) were conducted to evaluate performance stability across network complexities.

## 3. Results

Table 2 and Table 3 present the model evaluation results for white-light cystoscopy (RGB-WLC) and hyperspectral reconstructed narrow-band imaging, respectively. In this study, sensitivity was adopted as the primary evaluation metric to assess the ability of the models to detect lesion regions. Table 4 summarizes the performance comparison between the conventional white-light cystoscopy (WLC) model and the hyperspectral reconstructed narrow-band imaging model on the testing dataset (*N* = 415). The SAVE model achieved higher overall accuracy, sensitivity, and *F*1-*score* than the WLC model across all evaluated categories. Regarding overall performance, the SAVE model achieved an accuracy of 0.948 (95% CI: 0.927–0.969), compared with 0.852 (95% CI: 0.818–0.886) for the WLC model, representing a relative improvement of 11.27%. The difference was statistically significant (*p* < 0.001).

Category-specific performance is summarized as follows:

Normal bladder tissue (Normal): 

The SAVE model achieved sensitivity and precision values of 1.000 (*p* < 0.001). In comparison, the WLC model achieved a sensitivity of 0.740.

Early-stage bladder cancer lesions (Ta stage):

The sensitivity of the SAVE model increased from 0.846 to 0.923 (*p* = 0.034), while the *F*1-*score* improved from 0.717 to 0.889.

Muscle-invasive bladder cancer (Above T1):

The sensitivity of the SAVE model increased from 0.836 to 0.909 (*p* = 0.046), while the *F*1-*score* improved from 0.860 to 0.926.

Table 5 presents the prediction distribution of the hyperspectral reconstructed narrow-band imaging model across all categories in the testing dataset. Among the 415 testing images, 402 images were correctly classified as true positives (TP), whereas 13 images were classified as false negatives (FN). In addition, 15 images from other categories were incorrectly classified as false positives (FP). For the Normal category, all 50 images were correctly classified (TP = 50, F*N* = 0, FP = 0), resulting in sensitivity and precision values of 1.000. For the Ta category, 60 of 65 images were correctly classified, while five images were classified as false negatives and 10 images from other categories were incorrectly classified as Ta lesions. The resulting sensitivity and *F*1-*score* were 0.923 and 0.889, respectively.

For the Above T1 category, 50 of 55 images were correctly classified, while five images were classified as false negatives and three images from other categories were incorrectly classified as Above T1 lesions. The resulting sensitivity and *F*1-*score* were 0.909 and 0.926, respectively. Overall, the SAVE model achieved higher classification performance than the WLC model across all evaluated categories, including both Ta-stage and Above T1-stage lesions.

**Table 4:** Comparison of Core Metrics and Model Performance (Testing Dataset, *N* = 415).

Category	Imaging Model	*Sensitivity* (95% CI)	*Precision* (95% CI)	*F*1-*Score*	(*p*-Value)
Normal	WLC	0.740 (0.619–0.861)	0.949 (0.888–1.000)	0.832	<0.001 *
Normal	SAVE	1.000 (0.929–1.000)	1.000 (0.929–1.000)	1.000	<0.001 *
Ta	WLC	0.846 (0.758–0.934)	0.623 (0.505–0.741)	0.717	0.034 *
Ta	SAVE	0.923 (0.858–0.988)	0.857 (0.772–0.942)	0.889	0.034 *
Above T1	WLC	0.836 (0.738–0.934)	0.885 (0.801–0.969)	0.860	0.046 *
Above T1	SAVE	0.909 (0.833–0.985)	0.943 (0.882–1.000)	0.926	0.046 *
Accuracy	WLC	0.852 (0.818–0.886)			<0.001 *
Accuracy	SAVE	0.948 (0.927–0.969)			<0.001 *

Note: * indicates a statistically significant difference (*p* < 0.05). All performance metrics were obtained from the validation results of the testing dataset (*N* = 415).

**Table 5:** Prediction Distribution of the Hyperspectral Reconstructed Narrow-Band Imaging Model on the Testing Dataset (*N* = 415).

Category	Number of Actual Images (n)	True Predictions (TP)	False Negatives (FN)	False Positives (FP)	*Sensitivity*	*Precision*	*F*1-*Score*
Normal	50	50	0	0	1	1	1
Ta	65	60	5	10	0.923	0.857	0.889
Above T1	55	50	5	3	0.909	0.943	0.926
Hose	44	43	1	2	0.977	0.956	0.966
Loop	72	71	1	0	0.986	1	0.993
TURBT	129	128	1	0	0.992	1	0.996
Total	415	402	13	15	--	--	--

## 4. Discussion

### 4.1. Interpretation of Stage-Specific Performance

Overall, the results demonstrated that, in both imaging models, the Above T1 category achieved superior performance compared with the Ta category in terms of sensitivity, precision, and *F*1-*score*. This phenomenon can primarily be attributed to the fact that Ta-stage lesions often present as small papillary or flat structures with indistinct boundaries and insufficient contrast relative to surrounding normal tissues, making image recognition more challenging. In contrast, Above T1 tumors generally exhibit more prominent tumor morphology and clearer boundary characteristics, allowing them to be more readily identified by the models.

Further comparison between the two imaging modalities revealed that the sensitivity of the Ta category was 0.846 and 0.923 for the white-light cystoscopy model and the hyperspectral reconstructed narrow-band imaging model, respectively. Similarly, for the Above T1 category, the sensitivities were 0.836 and 0.909, respectively. These findings indicate that the hyperspectral reconstructed narrow-band imaging model consistently outperformed the conventional white-light cystoscopy model across all classification categories.

### 4.2. Impact of Hyperspectral Features on Lesion Recognition

**Figure 4:**
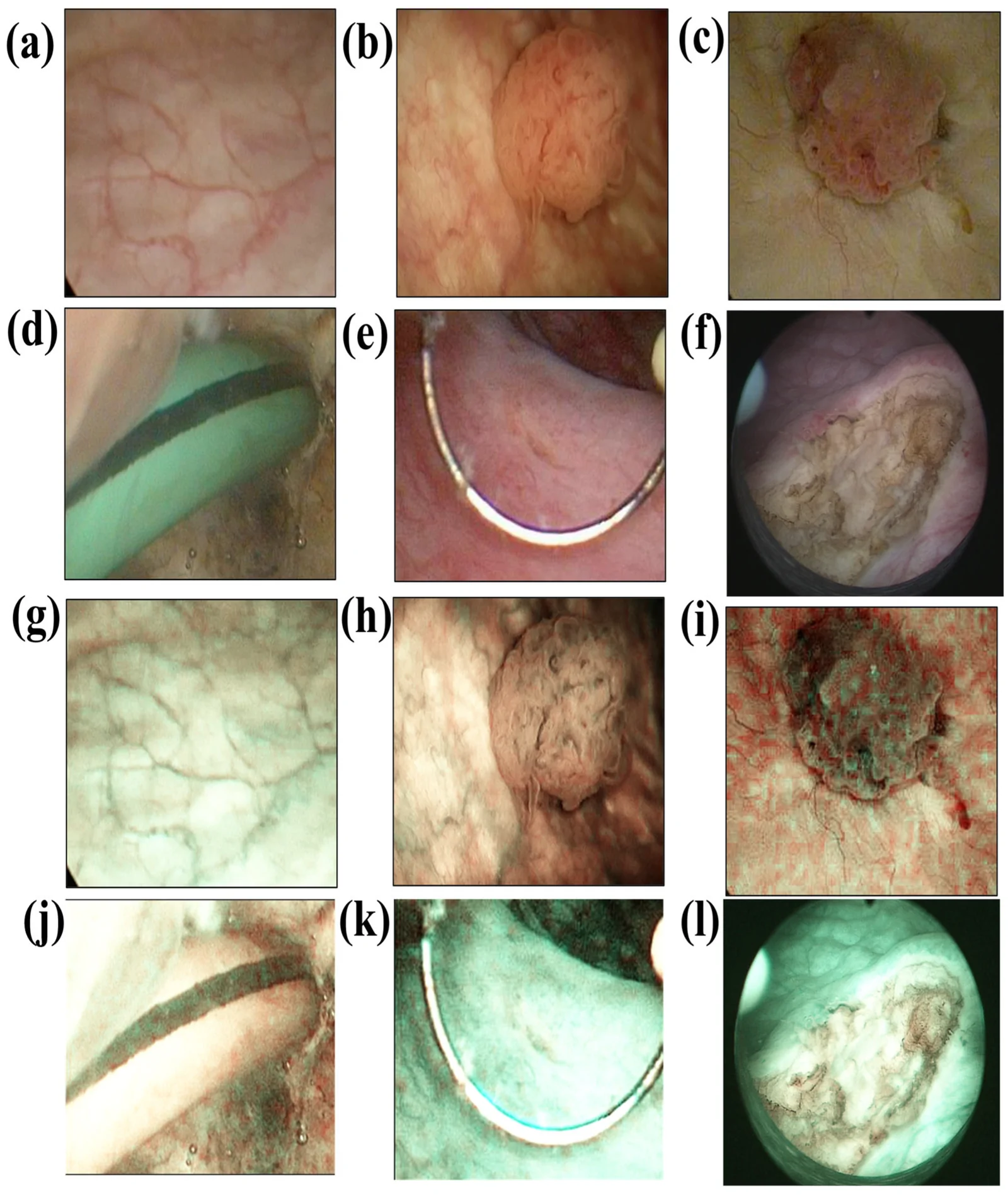
Representative white-light cystoscopy (WLC) and Spectrum-Aided Visual Enhancer (SAVE) reconstructed narrow-band images across all six target categories and typical misclassification cases. (**a**–**f**) Conventional WLC images and (**g**–**l**) corresponding SAVE reconstructed images for: (**a**,**g**) Normal bladder tissue; (**b**,**h**) Ta-stage tumor; (**c**,**i**) Above T1-stage tumor; (**d**,**j**) Irrigation Hose; (**e**,**k**) Resectoscope Loop; and (**f**,**l**) TURBT.

**Figure 5:**
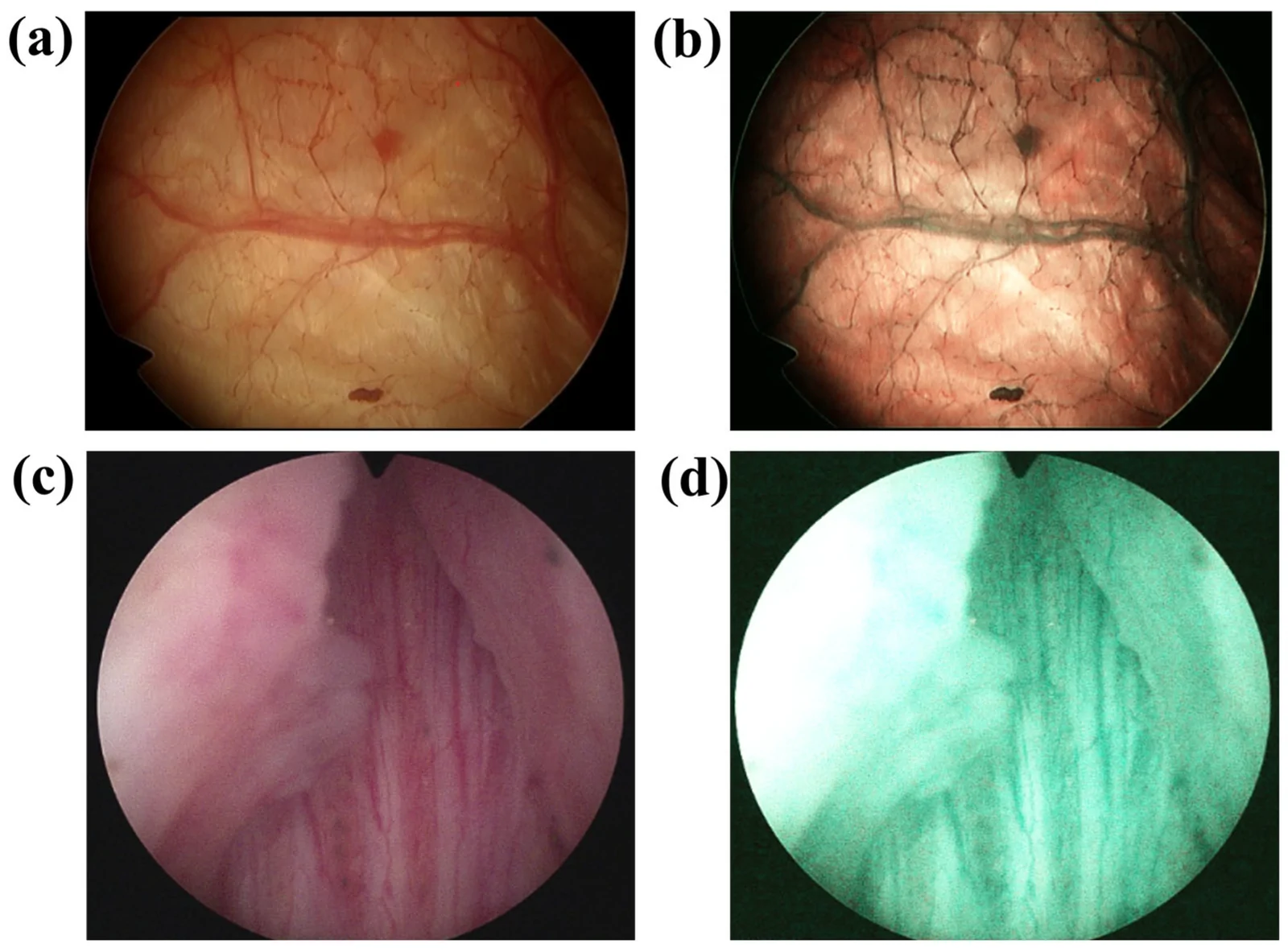
Representative white-light cystoscopy (WLC) and Spectrum-Aided Visual Enhancer (SAVE) reconstructed narrow-band images across typical misclassification cases. Representative misclassified cases under WLC and SAVE modalities: (**a**,**b**) False Negative (FN) case where an early Ta-stage tumor was misclassified as Normal mucosa due to low contrast under WLC, but demonstrated marked microvascular contrast enhancement after SAVE reconstruction; (**c**,**d**) False Positive (FP) case where Normal mucosal hyperemia was misclassified as a Ta-stage tumor due to localized capillary congestion.

The improved performance observed in the SAVE model may be associated with the additional spectral information provided by hyperspectral reconstruction. As shown in Figure 4, representative SAVE images exhibited enhanced visualization of vascular structures and improved lesion conspicuity compared with conventional white-light cystoscopy (WLC), particularly in Ta-stage lesions. These image characteristics may facilitate the identification of lesion-related features by the deep learning model. Analysis of misclassified cases revealed key clinical insights into model limitations and the additive value of SAVE hyperspectral reconstruction. As illustrated in Figure 5, False Negative (FN) errors predominantly occurred when subtle Ta-stage papillary tumors presented with flat morphology or low contrast against the background mucosa under conventional white light, leading to misclassification as Normal tissue. SAVE reconstruction effectively rectified such visual ambiguities by emphasizing subepithelial microvascular loops at 415 nm and 540 nm. Conversely, False Positive (FP) errors occurred when Normal mucosal regions with benign inflammatory hyperemia or focal capillary dilation mimicked early neoplastic angiogenesis, prompting false Ta-stage predictions. Integrating spectral feature representation helps discriminate true malignant neoangiogenesis from benign mucosal congestion.

It is worth highlighting the clinical necessity of incorporating non-pathological categories (Irrigation Hose, Resectoscope Loop, and TURBT) alongside pathological staging classes. In routine TURBT procedures, metallic electrodes, fluid tubing, and thermal cautery margins frequently occupy the endoscopic field of view. Incorporating these common surgical artifacts into model training ensures that the deep learning architecture learns to discriminate non-tissue elements from true urothelial neoplasms, thereby preventing false-positive predictions during real-world clinical deployment.

### 4.3. Comparison with Conventional and Narrow-Band Imaging Techniques

Compared with conventional white-light cystoscopy (WLC), advanced endoscopic imaging modalities such as narrow-band imaging (NBI) and blue-light cystoscopy (BLC) have been developed to improve lesion visualization and diagnostic accuracy. These techniques enhance the visibility of mucosal microvascular patterns and subtle tissue abnormalities that may be difficult to identify under standard white-light conditions. Consequently, NBI and BLI have demonstrated improved diagnostic performance for the detection of early-stage bladder lesions compared with conventional WLC.

In contrast, the SAVE approach proposed in this study reconstructs narrow-band spectral information directly from conventional white-light images without requiring additional hardware modifications. This characteristic provides a practical advantage for retrospective image analysis and facilitates integration into existing cystoscopic imaging workflows. Unlike NBI and BLI systems, which rely on dedicated optical filters and specialized imaging equipment, SAVE can be implemented using existing white-light image datasets, potentially reducing equipment costs and improving accessibility in routine clinical practice. The results of this study demonstrated that the SAVE model consistently outperformed the conventional WLC model in both bladder cancer classification and detection tasks. The improvement was particularly evident for Ta-stage lesions, where sensitivity increased from 0.846 to 0.923, and the *F*1-*score* increased from 0.717 to 0.889. The superior performance observed in this study suggests that SAVE can provide diagnostically relevant spectral information while maintaining compatibility with conventional white-light cystoscopy systems. Overall, hyperspectral reconstructed narrow-band imaging demonstrated superior performance compared with conventional white-light cystoscopy while maintaining compatibility with standard clinical imaging systems. Therefore, SAVE may represent a practical and cost-effective alternative for improving bladder cancer detection and supporting computer-aided diagnostic applications in clinical settings.

Furthermore, recent studies evaluating deep learning models on RGB cystoscopic images, such as Hwang et al. [62], reported classification accuracies reaching 0.912 for four-stage bladder cancer classification (CIS, Ta, T1, T2). In comparison, our baseline RGB-WLC model achieved an accuracy of 0.852. This difference is primarily attributable to task scope and dataset composition: while curated datasets focus strictly on cancer staging, our study evaluated a broader 6-class real-world dataset incorporating common surgical equipment and artifacts (Irrigation Hose, Resectoscope Loop, and TURBT) to mirror routine TURBT procedures. Notably, when enhanced by the SAVE hyperspectral reconstruction algorithm, our model achieved an overall accuracy of 0.948, significantly outperforming both the unenhanced WLC baseline (0.852, *p* < 0.001) and literature benchmarks, highlighting the efficacy of spectral feature enhancement in complex clinical environments.

Beyond urological applications, hyperspectral imaging (HSI) has emerged as a pivotal non-invasive modality in clinical oncology, demonstrating remarkable efficacy in intraoperative brain tumor delineation, head and neck cancer margin classification, and ovarian tissue characterization. These diverse applications highlight HSI’s capacity to resolve subtle physiological and spectro-morphological variations, thereby offering critical decision support and improving diagnostic accuracy across multiple solid tumor domains. The diagnostic superiority of the SAVE framework observed in this bladder cancer study aligns with previous clinical findings in cutaneous oncology. Specifically, Lin et al. demonstrated that converting conventional RGB images into pseudo-hyperspectral representations via SAVE significantly boosted the detection metrics (precision, recall, *F*1-*score*, and mAP) of YOLO architectures across various melanoma subtypes [63,64]. Extending the SAVE approach from surface dermatology to endourological cystoscopy further confirms its robust generalizability in extracting microvascular hemoglobin absorption features across distinct anatomical and clinical imaging environments. Our current findings significantly extend the preliminary benchmarks reported by Yang et al. [61], where the SAVE algorithm was evaluated using older-generation architectures such as VGG16, ResNet34, and YOLOv5. While their baseline study achieved promising targeted improvements (e.g., VGG16 improving the *F*1-*score* of the Above T1 category from 65% under WLI to 85% under SAVE), our current framework leveraging YOLOv8x-cls achieved a superior overall accuracy of 0.948 and an *F*1-*score* of 0.926 for the Above T1 stage. This comparison underlines that pairing the spectral-enhanced features of the SAVE algorithm with state-of-the-art real-time classification architectures (YOLOv8) yields a highly synergistic performance boost, particularly in resolving subtle mucosal tumors.

### 4.4. Ablation Study and Model Robustness

To investigate whether the performance improvement observed in this study was primarily attributable to the hyperspectral reconstructed narrow-band imaging approach rather than model complexity alone, an ablation study was conducted using YOLOv8 models with different parameter scales. In addition to the YOLOv8x-cls model, lightweight YOLOv8n-cls and YOLOv8s-cls models were also trained and evaluated using both the conventional white-light cystoscopy (WLC) dataset and the SAVE dataset.

**Figure 6:**
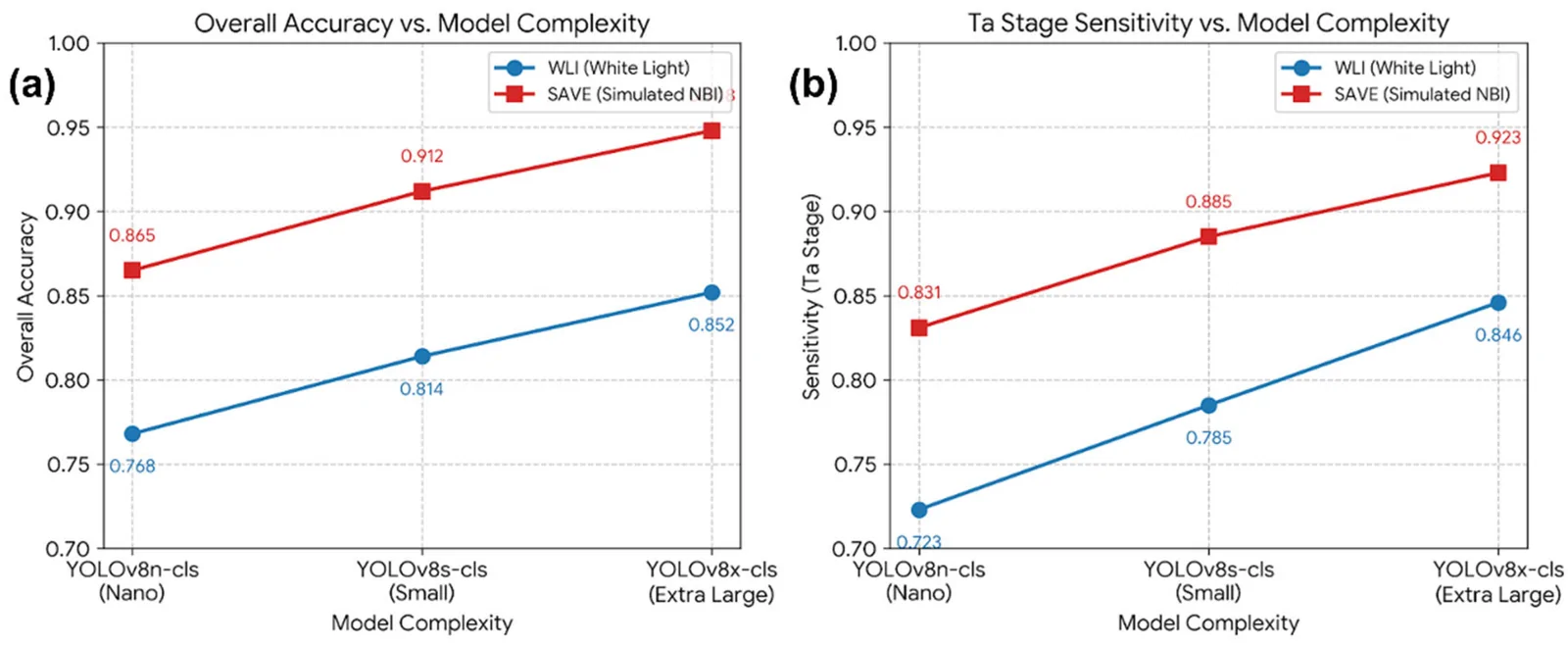
Performance Comparison Between Conventional White-Light Cystoscopy and Hyperspectral Reconstructed Narrow-Band Imaging Across Models with Different Complexity Levels. (**a**) Comparison of overall model accuracy across different YOLOv8 model scales. (**b**) Comparison of Ta-stage lesion sensitivity across different YOLOv8 model scales. The blue line represents conventional white-light cystoscopy (WLC), whereas the red line represents hyperspectral reconstructed narrow-band imaging.

Figure 6 and Table 6 present the performance comparison results across models with different levels of complexity. Across all model scales, the SAVE dataset consistently achieved higher overall accuracy and lesion sensitivity than the conventional WLC dataset. Notably, the lightweight YOLOv8n-cls model trained using SAVE images achieved an overall accuracy of 0.865, exceeding the accuracy of 0.852 obtained by the YOLOv8x-cls model trained using conventional WLC images. This finding suggests that the performance improvement observed in this study was primarily associated with the enhanced spectral information provided by the SAVE approach rather than increases in network complexity alone.

Further analysis of the sensitivity results for early-stage lesions revealed different responses to reduced model complexity between the two imaging modalities. When the model architecture was reduced from YOLOv8x-cls to YOLOv8n-cls, the sensitivity of the WLC model for Ta-stage lesions decreased from 0.846 to 0.723. In contrast, the SAVE model maintained a sensitivity of 0.831 under the YOLOv8n-cls configuration, remaining close to the performance of the large-scale WLC model. Similarly, for Above T1 lesions, the SAVE model preserved stable performance across different model scales, achieving a sensitivity of 0.840 under the lightweight Nano configuration.

Overall, the results demonstrate that the SAVE approach consistently outperformed conventional WLC across different parameter scales, including Nano, Small, and Extra Large architectures. These findings demonstrate the robustness of the hyperspectral features generated by the SAVE algorithm and suggest that the proposed approach may be suitable for deployment in resource-constrained clinical environments. Even when lightweight models were employed, SAVE maintained stable performance for bladder cancer recognition, particularly in the detection of early-stage lesions.

To further verify whether the performance enhancement of the SAVE framework was independent of the chosen deep learning architecture, a cross-architecture comparison was conducted using the standard ResNet-50 backbone alongside the YOLOv8 classification family (YOLOv8n, YOLOv8s, and YOLOv8x). As summarized in Table 6, ResNet-50 achieved an overall accuracy of 0.838 under conventional white-light cystoscopy (WLC), which increased to 0.928 when using SAVE hyperspectral reconstructed images (+9.0% improvement). Similarly, early-stage Ta lesion sensitivity improved from 0.815 to 0.908. The consistent diagnostic superiority of SAVE across both residual CNN architectures (ResNet-50) and modern real-time object classification frameworks (YOLOv8) demonstrates that the observed performance gains are architecture-agnostic. This confirms that the primary mechanism underlying the accuracy gain is the extraction of diagnostically relevant 415 nm and 540 nm spectral absorption features rather than specific network parameterization.

### 4.5. Clinical Implications and Study Limitations

The findings of this study have several potential clinical implications. Early detection of bladder cancer is essential for improving treatment outcomes and reducing disease progression. The improved sensitivity observed for Ta-stage lesions suggests that the SAVE approach may assist clinicians in identifying subtle early-stage tumors that are often difficult to recognize using conventional white-light cystoscopy. An important advantage of the proposed method is that SAVE images can be reconstructed directly from standard white-light cystoscopic images without requiring dedicated optical hardware. This characteristic may facilitate integration into existing clinical workflows, reduce equipment costs, and support the development of computer-aided diagnostic systems for bladder cancer screening and staging.

Despite these promising results, several limitations should be acknowledged. First, the study was conducted using a single-center dataset consisting of 1372 cystoscopic images obtained from 129 patients, which may limit the generalizability of the findings. Second, although the 30% hold-out dataset was evaluated without iterative hyperparameter tuning, the reliance on a single 7:3 train-test split rather than a prospective, multi-center three-way split (Train/Validation/Test) or k-fold cross-validation represents a methodological limitation. Third, variations in image quality, particularly in TURBT images, may have influenced model performance. Fourth, the hyperspectral reconstruction process utilized only the 415 nm and 540 nm wavelength bands. Additional wavelength combinations may provide further diagnostic information and should be investigated in future studies. Future work will incorporate strict three-way dataset partitioning and 5-fold cross-validation across independent institutions to provide further out-of-fold generalization bounds. In addition, further optimization of wavelength selection and integration with real-time cystoscopic systems may enhance the clinical applicability of the SAVE framework.

Fifth, benign bladder lesions (such as chronic cystitis, cystitis cystica, and benign urothelial papillomas) were not categorized as an independent training class in the current model architecture. Although the SAVE hyperspectral reconstruction at 415 nm and 540 nm enhances microvascular contrast to aid in differentiating malignant neoangiogenesis from normal mucosa, benign inflammatory lesions may occasionally present with overlapping visual features under white-light cystoscopy. In the current workflow, the proposed AI system functions as a computer-aided decision support tool, with definitive diagnosis confirmed by pathological biopsy. Future iterations of this framework will incorporate prospective, multi-center datasets containing explicitly labeled benign lesion classes to further improve model specificity and clinical robustness.

Seventh, it should be explicitly clarified that the proposed Spectrum-Aided Visual Enhancer (SAVE) framework represents a computational, software-defined optical sensing approach rather than a novel physical optical sensor hardware. While conventional biosensors rely on physical transducers or optical filter hardware to acquire biological signals, our framework algorithmically extracts tissue-specific hemoglobin absorption signatures (415 nm and 540 nm) from standard RGB white-light cystoscopic inputs. By bridging standard optical imaging and advanced mathematical spectral reconstruction, this approach achieves virtual narrow-band biosensing capabilities without requiring specialized hardware overhauls or camera sensor modifications.

## 5. Conclusions

This study developed and evaluated a bladder cancer recognition framework based on hyperspectral reconstructed narrow-band imaging and deep learning using 1372 cystoscopic images obtained from 129 patients. The proposed approach aimed to enhance lesion visualization through hyperspectral reconstruction and improve diagnostic performance for bladder cancer classification. The experimental results demonstrated that the SAVE model consistently outperformed the conventional white-light cystoscopy (WLC) model across all evaluated categories. Compared with WLC, SAVE achieved higher overall accuracy, sensitivity, and *F*1-*score*, with the most notable improvement observed in Ta-stage lesions. Furthermore, the ablation study showed that the performance advantages of SAVE were maintained across different YOLOv8 model scales, indicating the robustness of the reconstructed spectral features. Overall, this study successfully demonstrates the powerful synergy between advanced optical biosensing and modern AI processing techniques, thereby expanding the boundaries of medical imaging. The proposed SAVE framework provides a practical approach for enhancing lesion recognition while maintaining full compatibility with conventional cystoscopic imaging systems. By bridging the gap between complex optical data and automated interpretation, this integrated paradigm offers promising new possibilities for future smart healthcare and clinical decision support systems. Future studies involving larger multi-center datasets, external validation cohorts, and real-time cystoscopic implementation will further enhance the generalizability and clinical applicability of this framework, successfully propelling the future of automated healthcare technologies.

## Figures and Tables

**Table 1:** Dataset Distribution of the Hyperspectral Biosensing Model (SAVE).

Category/Subtype	Training Set (70%)		Testing Set (30%)	
	Cases	Images	Cases	Images
Normal	50	116	20	50
Ta Stage	16	146	7	65
Above T1 Stage	34	124	16	55
Irrigation Hose (formerly “Hose”)	N/A	102	N/A	44
Resectoscope Loop (formerly “Loop”)	N/A	168	N/A	72
TURBT	N/A	301	N/A	129
Total	100 *	957	43 *	415

* Notes: Cases refer to distinct diagnostic category occurrences across 129 unique clinical patients. A single patient may contribute to multiple categories (e.g., presenting both tumor lesions and normal tissue). “Irrigation Hose” (formerly “Hose”) and “Resectoscope Loop” (formerly “Loop”) represent surgical equipment and artifact categories. Subtypes (Papillary Ta, Flat Ta/CIS, T1, T2+) illustrate histological dataset composition.

**Table 2:** Performance Metrics of the White-Light Cystoscopy (RGB-WLC) Detection Model.

	Metric	*Sensitivity*	*Precision*	*F*1-*Score*	*Accuracy*
Category					
Normal (*n* = 50)		0.740	0.949	0.832	0.852
Ta (*n* = 65)		0.846	0.623	0.717	
Above T1 (*n* = 55)		0.836	0.885	0.860	
Hose (*n* = 44)		0.886	0.951	0.917	
Loop (*n* = 72)		1.000	0.987	0.993	
TURBT (*n* = 129)		1.000	0.987	0.993	

**Table 3:** Performance Metrics of the Hyperspectral Reconstructed Narrow-Band Imaging Detection Model.

	Metric	*Sensitivity*	*Precision*	*F*1-*Score*	*Accuracy*
Category					
Normal (*n* = 50)		1.000	1.000	1.000	0.948
Ta (*n* = 65)		0.923	0.857	0.889	
Above T1 (*n* = 55)		0.909	0.943	0.926	
Hose (*n* = 44)		0.977	0.956	0.966	
Loop (*n* = 72)		0.986	1.000	0.993	
TURBT (*n* = 129)		0.992	1.000	0.996	

**Table 6:** Performance Comparison Across Different Model Complexities.

Training Model	Model Complexity (Parameter Scale)	Imaging Modality	Overall Accuracy	Ta-Stage Sensitivity	Above T1-Stage Sensitivity
ResNet-50	Standard Residual CNN	WLC	0.838	0.815	0.818
		SAVE	0.928	0.908	0.891
YOLOv8n-cls	Nano	WLC	0.768	0.723	0.745
		SAVE	0.865	0.831	0.84
YOLOv8s-cls	Small	WLC	0.814	0.785	0.792
		SAVE	0.912	0.885	0.876
YOLOv8x-cls	Extra Large	WLC	0.852	0.846	0.836
		SAVE	0.948	0.923	0.909

## Data Availability

The data presented in this study are available on request from the corresponding author. The data are not publicly available due to strict privacy and ethical restrictions mandated by the Institutional Review Board of Dalin Tzu Chi Hospital (B11403015) and the Institutional Review Board of Kaohsiung Armed Forces General Hospital (KAFGHIRB 115-014).

## Supplementary Materials

The following supporting information can be downloaded at: https://www.mdpi.com/article/10.3390/bios16080445/s1. Figure S1 Workflow for establishing the relationship matrix between the spectrometer and the cystoscope. Figure S2 Illumination spectra measured using the spectrometer. (a) White-light endoscopic illumination spectrum. (b) Narrow-band endoscopic illumination spectrum. Figure S3 XYZ color-matching functions. Figure S4 Polynomial regression plots for the white-light cystoscope. (a) First-order polynomial regression curve for the white-light cystoscope. (b) Second-order polynomial regression curve for the white-light cystoscope. (c) Third-order polynomial regression curve for the white-light cystoscope. (d) Fourth-order polynomial regression curve for the white-light cystoscope. Figure S5 Polynomial regression plots for the narrow-band cystoscope. (a) First-order polynomial regression curve for the narrow-band cystoscope. (b) Second-order polynomial regression curve for the narrow-band cystoscope. (c) Third-order polynomial regression curve for the narrow-band cystoscope. (d) Fourth-order polynomial regression curve for the narrow-band cystoscope. Figure S6 The first 12 principal components of R_Spectrum_. Figure S7 Spectral differences for six selected color patches under white-light cystoscopy. Figure (a)–(f) correspond to color patches 13–18, respectively, among the 24 color patches listed in Table S3 for the white-light root-mean-square-error analysis. Figure S8 Spectral differences for six selected color patches under narrow-band cystoscopy. Figure (g)–(l) correspond to color patches 13–18, respectively, among the 24 color patches listed in Table S3 for the narrow-band root-mean-square-error analysis. Table S1. Root-mean-square errors of the 24 color patches under white-light and narrow-band cystoscopy. Table S2. Color-difference comparison between the measured and simulated spectra of the 24 color patches under white-light cystoscopy. Table S3. Color-difference comparison between the measured and simulated spectra of the 24 color patches under narrow-band cystoscopy.
