# Supplementary material for: Deep Learning-Assisted Accuracy Improvement in Bladder Cancer Staging of Spectrum-Aided Visual Enhanced Cystoscopy Images
Source: Biosensors (Basel). 2026 Aug 16;16(8):445. doi: 10.3390/bios16080445 (PMC13511581; doi:10.3390/bios16080445)
Supplement: Supplementary file 1 [file biosensors-16-00445-s001.zip › biosensors-4427433-supplementary.pdf]

1. Hyperspectral conversion procedures and algorithms

S1 Hyperspectral Transformation Workflow

The workflow of this study is shown in Figure S1. To establish an identical transformation relationship between the cystoscope (Stryker 4.0 mm 12° IR Filtered Autoclavable Cystoscope) and the spectrometer (Ocean Optics QE65000) within the visible-light range of 380–780 nm, both devices must be referenced to the same standard. Specifically, the cystoscope and spectrometer were used to acquire measurements of a standard 24-color chart (X-Rite ColorChecker Classic, 24 patches) in a dark-room environment in the operating room. The chromaticity values and spectral values obtained from the two devices were recorded, and a transformation matrix between them was calculated. This procedure ensured consistency between the measurements obtained using the cystoscope and the spectrometer within the visible-light range.

S1.1 Relationship Matrix Between Cystoscope $XYZ_{\text{Endoscope}}$ and Spectrometer $XYZ_{\text{Endoscope}}$

SpectraSuite software and a spectrometer were used to measure the spectra of the white-light cystoscope illumination source and the narrow-band cystoscope illumination source over the visible-light range of 380–780 nm at 1-nm intervals. The illumination spectra are shown in Figure S2. Images of the 24 color patches were acquired using the cystoscope in 8-bit sRGB format, while the corresponding spectral data of the 24 patches were obtained using the spectrometer. The data were subsequently converted into the CIE 1931 XYZ color space.

For the cystoscope, the image data were stored according to the sRGB color-space specification. Therefore, the individual R, G, and B values first had to be normalized

from the range of 0–255 to the range of 0–1. The gamma function shown in Equation S1 was then used to convert the original sRGB values into linear RGB values.

$$f(n) = \begin{cases} \left(\frac{n+0.055}{1.055}\right)^{2.4}, & n > 0.04045 \\ \left(\frac{n}{12.92}\right), & otherwise \end{cases} \tag{S1}$$

The linear RGB values were then converted into the CIE 1931 XYZ color space using the following equations:

$$\begin{bmatrix} X \\ Y \\ Z \end{bmatrix} = [M_A][T] \begin{bmatrix} f(R_{sRGB}) \\ f(G_{sRGB}) \\ f(B_{sRGB}) \end{bmatrix} \times 100\,,\ 0 \le \begin{matrix} R_{sRGB} \\ G_{sRGB} \\ B_{sRGB} \end{matrix} \le 1 \tag{S2}$$

$$[T] = \begin{bmatrix} 0.4104\ 0.3576\ 0.1805 \\ 0.2126\ 0.7152\ 0.0722 \\ 0.0193\ 0.1192\ 0.9505 \end{bmatrix} \tag{S3}$$

Equation S2 and the transformation matrix Tin Equation S3 were used to convert the linear RGB values into the XYZ color space. Because the white point of the D65 illuminant specified in the sRGB color space differs from the white point of the measured illumination source, the chromatic adaptation matrix $M_A$in Equation S4 was used to transform the D65 white point (X_{CW}, Y_{CW}, Z_{CW})into the white point of the measured illumination source (X_{SW}, Y_{SW}, Z_{SW}). This transformation yielded the actual XYZ values under the measured illumination conditions, denoted as $XYZ_{\mathrm{Endoscope}}$.

$$[M_A] = \begin{bmatrix} X_{SW}/X_{CW} & 0 & 0 \\ 0 & Y_{SW}/Y_{CW} & 0 \\ 0 & 0 & Z_{SW}/Z_{CW} \end{bmatrix} \tag{S4}$$

For the spectrometer, converting visible-light reflectance spectral data acquired from 380 to 780 nm at 1-nm intervals into the XYZ color space required the XYZ color-

matching functions $\bar{x}(\lambda)$, $\bar{y}(\lambda)$, and $\bar{z}(\lambda)$, as shown in Figure S3, together with the illumination spectrum $S(\lambda)$used during cystoscopic imaging.

Because the Y value in the XYZ color space is proportional to luminance, Equation S5 was used to calculate the normalization factor k. The upper limit of Y was normalized to 100 to obtain a standardized luminance scale.

$$k = 100/\int_{380nm}^{780nm} S(\lambda)\,\bar{y}(\lambda)d\lambda \tag{S5}$$

Equations S6–S8 were then used to convert the reflectance spectral data into the XYZ values specified in the XYZ color space, denoted as XYZ_{Spectrum}.

$$X = k\int_{380nm}^{780nm} S(\lambda)\,R(\lambda)\bar{x}(\lambda)d\lambda \tag{S6}$$

$$Y = k\int_{380nm}^{780nm} S(\lambda)\,R(\lambda)\bar{y}(\lambda)d\lambda \tag{S7}$$

$$Z = k\int_{380nm}^{780nm} S(\lambda)\,R(\lambda)\bar{z}(\lambda)d\lambda \tag{S8}$$

S1.2 Correction Matrix C

After $XYZ_{\text{Endoscope}}$and XYZ_{Spectrum}were obtained using the above transformation equations, several factors that could introduce errors into the cystoscopic images had to be considered when calculating the transformation relationship between $XYZ_{\text{Spectrum}}$and $XYZ_{\text{Endoscope}}$. These factors included the nonlinear response of the cystoscope, dark current, inaccurate color separation by the color filters, and color shift.

Therefore, multiple regression analysis was performed to obtain the correction coefficient matrix C used to calibrate the cystoscope, as shown in Equation S9.

$$[C] = [XYZ_{Spectrum}] \times pinv([V]) \qquad \text{(S9)}$$

For the nonlinear response of the cystoscope, the spectrometer was assumed to have a linear response. Color patches 19–24 of the standard 24-color chart, which represent variations in grayscale, were measured using both the cystoscope and the spectrometer. Equations S2 and S6–S8 were used to calculate $XYZ_{\text{Endoscope}}$ and XYZ_{Spectrum}, respectively, and linear regression analysis was subsequently performed.

The polynomial regression results for the white-light and narrow-band cystoscopes are shown in Figures S4 and S5, respectively. These results confirmed that the cystoscope exhibited a nonlinear response. For third-order regression, the coefficients of determination, R^2, for white-light imaging and narrow-band imaging were 0.9953 and 0.9607, respectively, indicating a high degree of agreement. Therefore, the nonlinear response of the cystoscope was assumed to be correctable using a third-order equation. The nonlinear-response correction variable, $V_{\text{Non-linear}}$, was defined as shown in Equation S10.

$$V_{Non-linear} = [X^3\ Y^3\ Z^3\ X^2\ Y^2\ Z^2\ X\ Y\ Z\ 1\ ]^T \qquad \text{(S10)}$$

For the camera dark current, the dark-current value was assumed to remain constant and not vary with the amount of incident light. Therefore, a constant term was used to represent the contribution of dark current, and the dark-current correction variable V_{Dark} was defined as shown in Equation S11.

$$V_{Dark} = [\alpha] \quad \text{(S11)}$$

The color-filter and color-shift components were related to errors in color matching. Because the images had already been converted into the XYZ color space, and because the XYZ color-matching functions $\bar{x}$, $\bar{y}$, and $\bar{z}$ are correlated in the spectral domain, V_{Color} was defined to include all possible combinations of the X, Y, and Z values, as shown in Equation S12.

$$V_{Color} = [XYZ\ XY\ YZ\ XZ\ X\ Y\ Z\ ]^T \quad \text{(S12)}$$

After all error factors were considered, the variable matrix V in Equation S9 was constructed by multiplying V_{Color}, used as the basis, by $V_{\text{Non-linear}}$. To prevent overcorrection, the result was limited to terms of no higher than the third order, and V_{Dark} was also included, as shown in Equation S13.

$$V = [X^3\ Y^3\ Z^3\ X^2Y\ X^2Z\ Y^2Z\ XY^2\ XZ^2\ YZ^2\ XYZ\ X^2\ Y^2\ Z^2\ XY\ XZ\ YZ\ X\ Y\ Z\ \alpha]^T \quad \text{(S13)}$$

After the correction matrix C was obtained using Equation S9, the $[X\ Y\ Z]^T$ matrix of $XYZ_{\text{Endoscope}}$ was expanded into the variable matrix V. Equation S14 was then used to calculate the corrected X, Y, and Z values, denoted as XYZ_{Correct}.

The root-mean-square errors between XYZ_{Correct} and XYZ_{Spectrum} were 1.40 for the white-light cystoscope and 2.39 for the narrow-band cystoscope. These results indicated that only minor differences existed between the spectra transformed from the cystoscopic images and the spectra measured using the spectrometer.

$$[XYZ_{Correct}] = [C] \times [V] \quad \text{(S14)}$$

S1.3 Color Difference

Because the wavelength range specified in this study was the visible-light range of 380–780 nm, the calibration performance of the cystoscope could be evaluated using color difference. The CIEDE2000 method was used to calculate color differences.

This method accounts for differences in the sensitivity of human vision to different colors and incorporates lightness compensation, chroma compensation, hue compensation, a hue-rotation correction term, and neutral-color compensation. These adjustments reduce discrepancies caused by variations in human color perception.

To calculate the CIEDE2000 color difference, XYZ_{Correct} and XYZ_{Spectrum} were first converted from the XYZ color space into the CIELAB color space using Equations S15–S18.

$$L^* = 116f\left(\frac{Y}{Y_n}\right) - 16 \tag{S15}$$

$$a^* = 500\left[f\left(\frac{X}{X_n}\right) - f\left(\frac{Y}{Y_n}\right)\right] \tag{S16}$$

$$b^* = 200\left[f\left(\frac{Y}{Y_n}\right) - f\left(\frac{Z}{Z_n}\right)\right] \tag{S17}$$

$$f(n) = \begin{cases} n^{\frac{1}{3}} & , \ n > 0.008856 \\ 7.787n + 0.137931 & , \ otherwise \end{cases} \tag{S18}$$

The CIEDE2000 calculation showed substantial color differences between the cystoscope and the spectrometer before calibration. For the white-light cystoscope, the calibration results are shown in Table S1. The mean color difference decreased from 12.79 before calibration to 2.99 after calibration.

For the narrow-band cystoscope, the calibration results are shown in Table S2. The

mean color difference decreased from 11.28 before calibration to 3.95 after calibration. Thus, color calibration substantially reduced the mean color differences for both white-light and narrow-band imaging.

S1.4 Transformation Matrix M

After the XYZ values obtained from the cystoscopic and spectroscopic data had been calibrated and validated using color-difference and root-mean-square-error analyses, the transformation matrix M was determined using the calibrated XYZ values of the 24 color patches obtained from the cystoscope, XYZ_{Correct}, and the reflectance spectra of the 24 color patches measured using the spectrometer, R_{Spectrum}.

Principal component analysis was performed on the spectral data of the 24 color patches, R_{Spectrum}, to identify the major components in the reflectance spectral data. The corresponding principal component scores were then subjected to multiple regression analysis together with the calibrated XYZ values of the 24 color patches, XYZ_{Correct}, to obtain the transformation matrix M.

As shown in Figure S6, the first 12 principal components explained 99.99% of the variance in the R_{Spectrum} data. Therefore, the first 12 principal components, denoted as EV, were used for dimensionality reduction to obtain the principal component scores, which were also referred to as eigenvalues in this study.

These scores were used in regression analysis with XYZ_{Correct}. The variables in V_{Color} were selected for the multiple regression analysis because V_{Color} included all possible combinations of X, Y, and Z. The transformation matrix M was calculated using Equation S19. The simulated spectrum, S_{Spectrum}, was then calculated from XYZ_{Correct} using Equations S12 and S20.

$$[M] = [Score] \times pinv\ \ ([V_{Color}]) \tag{S19}$$

$$[S_{Spectrum}]_{380-780nm} = [EV][M][V_{Color}] \tag{S20}$$

The simulated spectra of the 24 color patches, S_{Spectrum}, were subsequently compared with the measured reflectance spectra, R_{Spectrum}, by evaluating the root-mean-square error and color difference for each patch.

Figures S7 and S8 show the spectral differences for six selected color patches under white-light and narrow-band cystoscopy, respectively. Table S3 summarizes the root-mean-square errors of the 24 color patches. The mean root-mean-square error was 0.058 for the white-light cystoscope and 0.096 for the narrow-band cystoscope.

Tables S4 and S5 compare the measured and simulated chromaticity results of the 24 color patches under white-light and narrow-band cystoscopy, respectively. The mean color difference was 2.99 for the white-light cystoscope, as calculated from Table S4, and 3.95 for the narrow-band cystoscope, as calculated from Table S5.

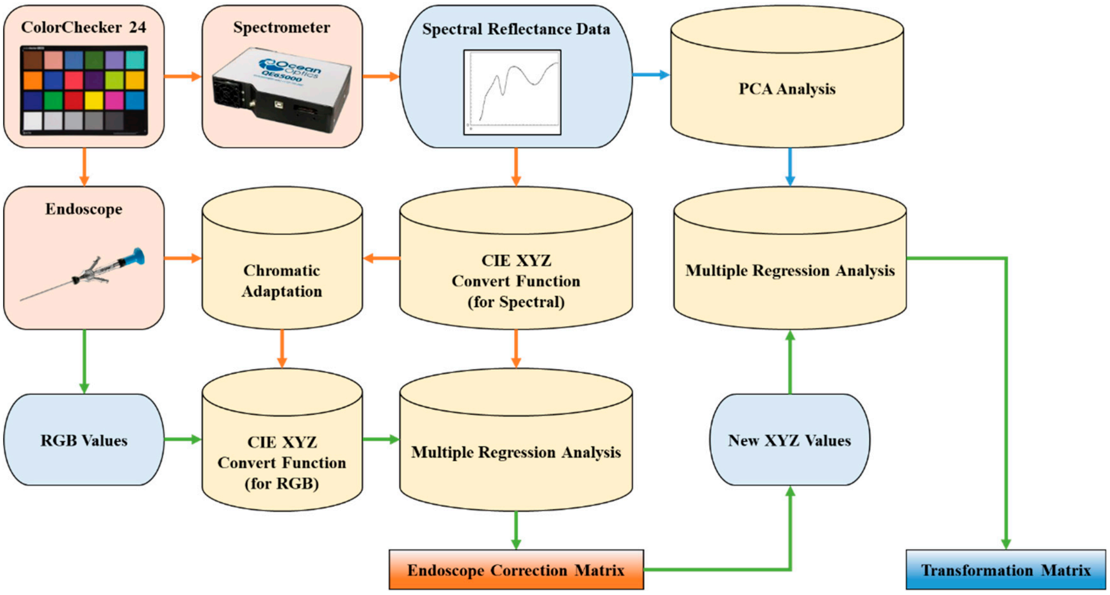

Figure S1 Workflow for establishing the relationship matrix between the spectrometer and the cystoscope.

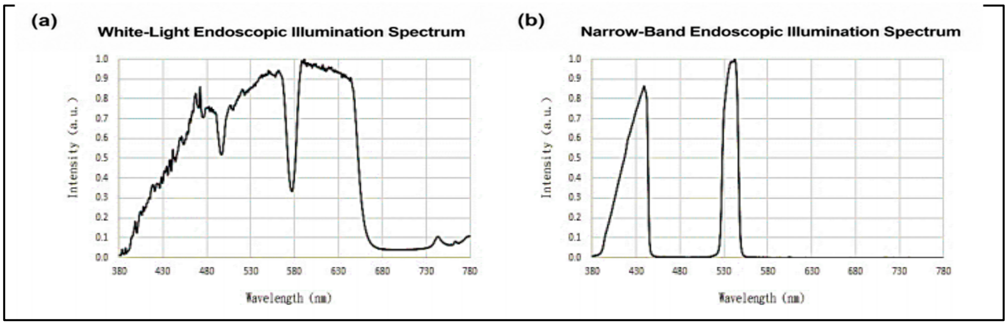

Figure S2 Illumination spectra measured using the spectrometer. (a) White-light endoscopic illumination spectrum. (b) Narrow-band endoscopic illumination spectrum.

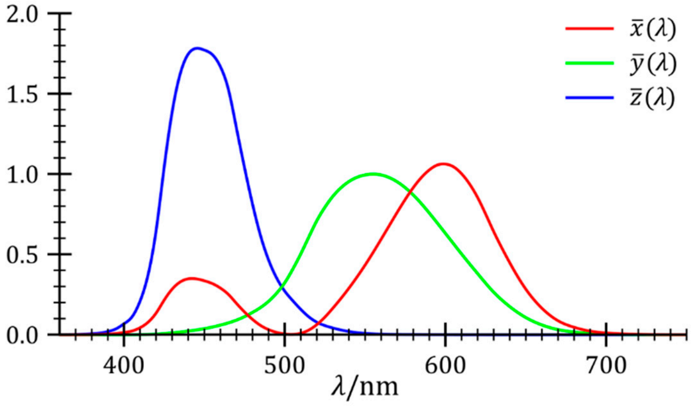

Figure S3 XYZ color-matching functions.

(a)

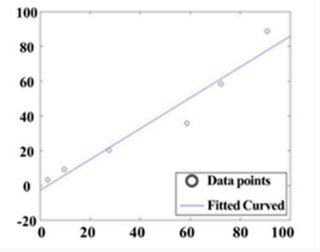

Linear regression equation

$$f(x) = 0.88719x - 2.791$$

Coefficient of determination:

$$R^2 = 0.9366$$

(b)

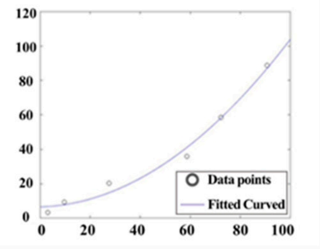

Second-order polynomial regression equation

$$f(x) = 0.0093221x^2 - 0.041724x + 6.536$$

Coefficient of determination:

$$R^2 = 0.9858$$

(c)

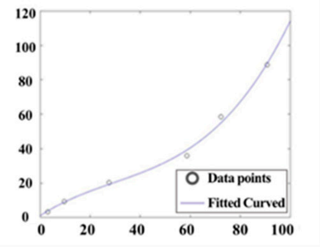

Third-order polynomial regression equation

$$f(x) = 0.00016608x^3 - 0.014686x^2 + 0.94014x + 1.0395$$

Coefficient of determination:

$$R^2 = 0.9953$$

(d)

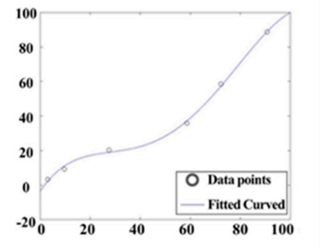

Fourth-order polynomial regression equation

$$f(x) = -5.9283 \times 10^{-6}x^4 - 0.0012739x^3 - 0.076438x^2 + 2.16849x - 3.67677$$

Coefficient of determination:

$$R^2 = 0.9984$$

Figure S4 Polynomial regression plots for the white-light cystoscope.

(a) First-order polynomial regression curve for the white-light cystoscope.

(b) Second-order polynomial regression curve for the white-light cystoscope.

(c) Third-order polynomial regression curve for the white-light cystoscope.

(d) Fourth-order polynomial regression curve for the white-light cystoscope.

(a)

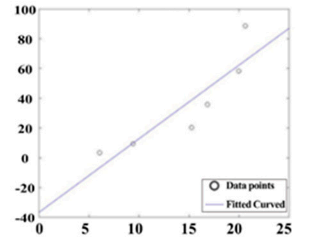

Linear regression equation

$$f(x) = 4.9474x - 36.7106$$

Coefficient of determination:

$$R^2 = 0.7873$$

(b)

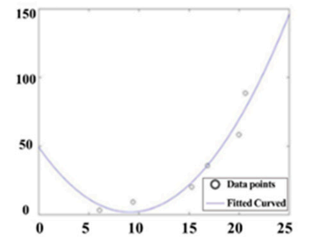

Second-order polynomial regression equation

$$f(x) = 0.57118x^2 - 10.4024x + 49.3272$$

Coefficient of determination:

$$R^2 = 0.9381$$

(c)

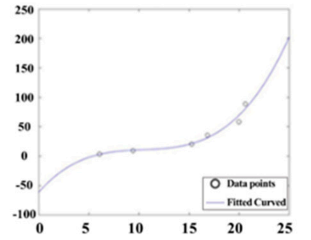

Third-order polynomial regression equation

$$f(x) = 0.057833x^3 - 1.7985x^2 + 19.3936x - 61.3077$$

Coefficient of determination:

$$R^2 = 0.9607$$

(d)

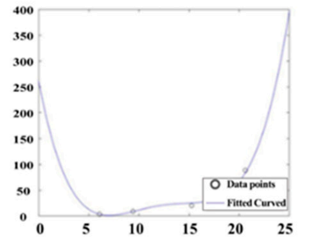

$$f(x) = 0.016414x^4 - 0.8024x^3 + 14.1072x^2 - 102.2269x + 261.446$$

Coefficient of determination:

$$R^2 = 0.9692$$

Figure S5 Polynomial regression plots for the narrow-band cystoscope.

(a) First-order polynomial regression curve for the narrow-band cystoscope.

(b) Second-order polynomial regression curve for the narrow-band cystoscope.

(c) Third-order polynomial regression curve for the narrow-band cystoscope.

(d) Fourth-order polynomial regression curve for the narrow-band cystoscope.

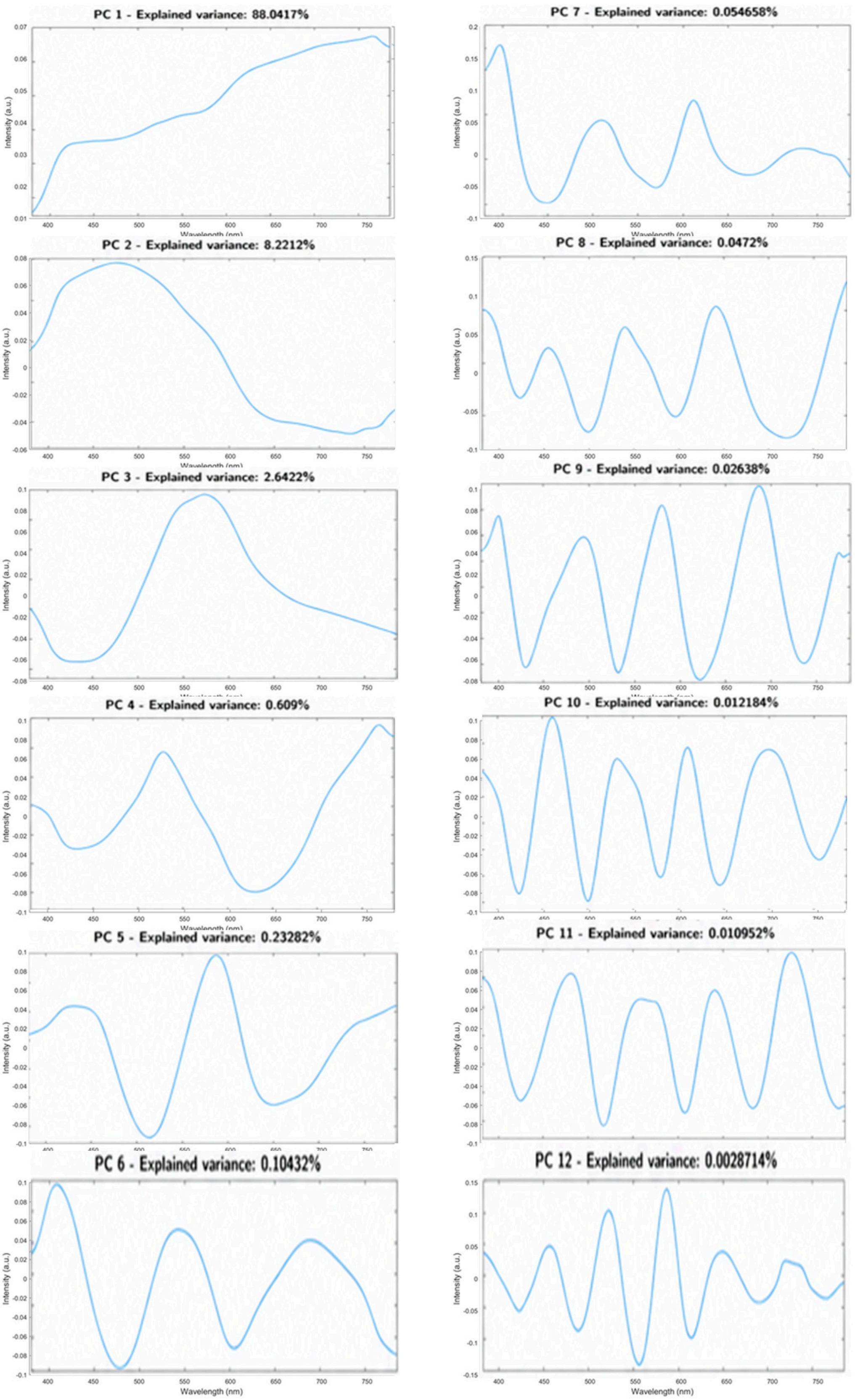

Figure S6 The first 12 principal components of $R_{Spectrum}$.

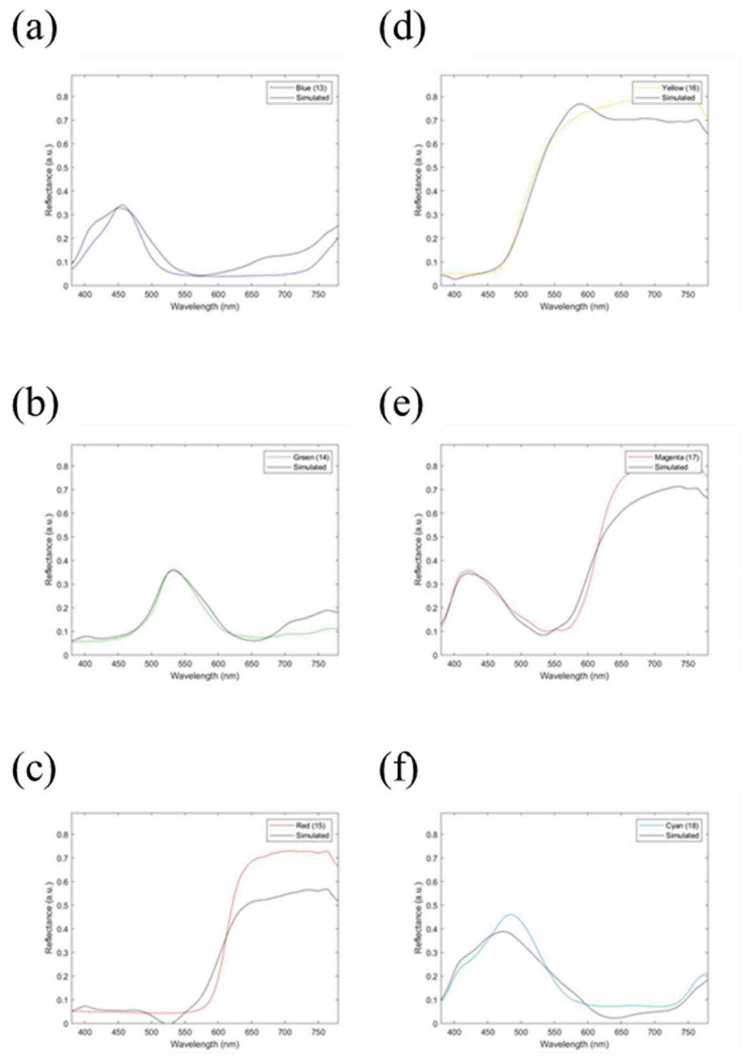

Figure S7 Spectral differences for six selected color patches under white-light cystoscopy. Figure (a)–(f) correspond to color patches 13–18, respectively, among the 24 color patches listed in Table S3 for the white-light root-mean-square-error analysis.

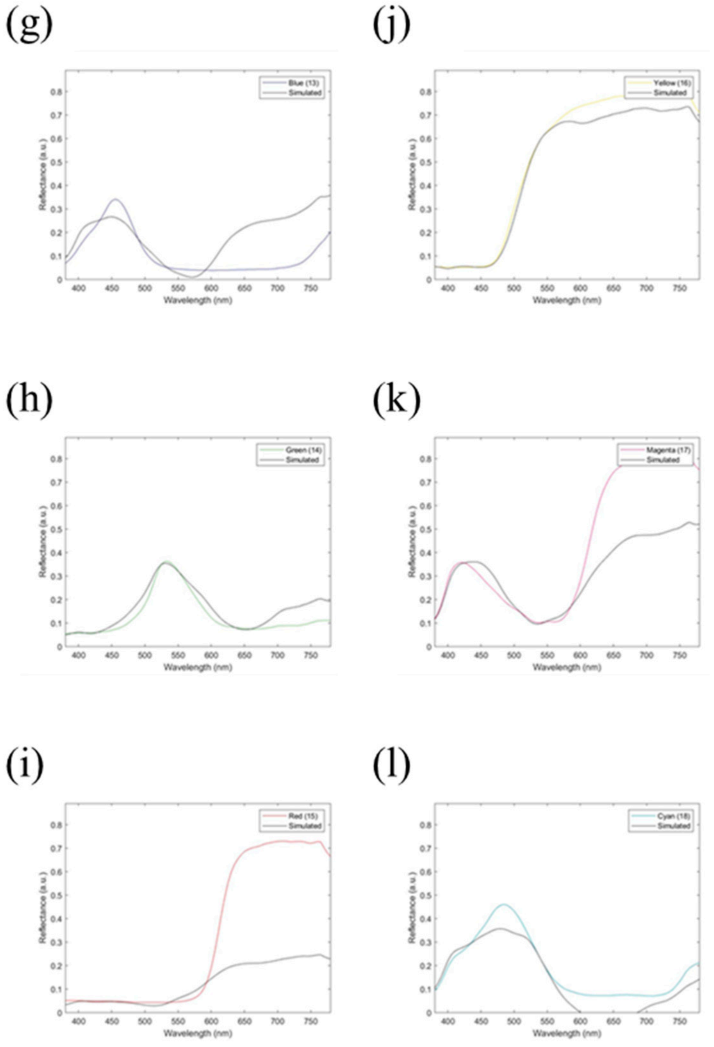

Figure S8 Spectral differences for six selected color patches under narrow-band cystoscopy. Figure (g)–(l) correspond to color patches 13–18, respectively, among the 24 color patches listed in Table S3 for the narrow-band root-mean-square-error analysis.

Table S1. Root-mean-square errors of the 24 color patches under white-light and narrow-band cystoscopy.

White-Light Root Mean Square Error			Narrowband Root Mean Square Error		
Color Patch Number	Figure Number	RMSE	Color Patch Number	Figure Number	RMSE
1	-	0.043	1	-	0.060
2	-	0.061	2	-	0.165
3	-	0.099	3	-	0.181
4	-	0.063	4	-	0.094
5	-	0.072	5	-	0.068
6	-	0.061	6	-	0.018
7	-	0.089	7	-	0.018
8	-	0.031	8	-	0.125
9	-	0.065	9	-	0.149
10	-	0.107	10	-	0.081
11	-	0.016	11	-	0.030
12	-	0.054	12	-	0.136
13	(a)	0.064	13	(g)	0.133
14	(b)	0.032	14	(h)	0.041
15	(c)	0.108	15	(i)	0.301
16	(d)	0.057	16	(j)	0.041
17	(e)	0.075	17	(k)	0.193
18	(f)	0.042	18	(l)	0.071
19	-	0.013	19	-	0.073
20	-	0.034	20	-	0.058
21	-	0.103	21	-	0.059
22	-	0.054	22	-	0.106
23	-	0.034	23	-	0.073
24	-	0.020	24	-	0.038
**Mean Error**		0.058	**Mean Error**		0.096

Panels (a)–(f) correspond to the spectral differences of the six color patches shown in Figure S7, whereas panels (g)–(l) correspond to the spectral differences of the six color patches shown in Figure S8.

Table S2. Color-difference comparison between the measured and simulated spectra of the 24 color patches under white-light cystoscopy.

Measured Spectrum			Measured 24 Color Patches	Simulated Spectrum			Simulated 24 Color Patches	Color Difference
L*	a*	b*		L*	a*	b*		
37.818	16.21	26.72		42.451	14.75	28		4.2
66.98	19.47	34.81		66.851	19.25	33.76		0.45
50.251	-5.29	-3.61		52.839	-4.88	-4.43		2.68
42.81	-14.15	32.29		45.572	-16.15	38.38		3.55
55.984	7.65	-4.84		55.537	5.67	-3.89		2.12
70.597	-31.32	19.18		70.507	-32.06	18.7		1.3
62.535	37.2	69.47		62.682	37.18	72.77		1.05
39.924	6.97	-24.32		34.949	12.38	-30.25		5.06
53.002	49.99	32.29		51.779	50.99	31.82		1.19
30.563	19.08	-7.07		35.762	15.43	3.66		8.83
72.198	-21.26	68.68		71.106	-21.52	69.46		1.03
72.881	21.95	78.35		72.672	22.06	76.34		0.45
28.837	14.18	-35.34		33.942	8.48	-28.99		4.91
55.4	-39.28	44.59		56.337	-37.48	43.74		1.03
42.844	56.12	38.68		43.016	55.88	36.6		0.87
82.595	7	90.53		82.767	6.93	90.52		0.2
52.083	48.96	5.52		52.5	48.7	5.85		0.66
50.315	-29.63	-10.47		49.213	-29.83	-10.47		1.29
95.468	1.26	26.02		95.717	1.19	25.89		2.5
80.963	1.38	22.33		79.157	1.83	22.81		2.22
66.385	1.05	18.85		69.526	1.62	20.02		1.73
52.187	0.88	15.6		46.881	2.5	11.85		6.43
36.468	0.33	11.65		30.848	3.41	4.48		8.76
21.4	0.21	7.8		18.589	0.04	-2.54		9.18
							Mean Color Difference	2.99

Table S3. Color-difference comparison between the measured and simulated spectra of the 24 color patches under narrow-band cystoscopy.

Measured Spectrum			Measured 24 Color Patches	Simulated Spectrum			Simulated 24 Color Patches	Color Difference
L*	a*	b*		L*	a*	b*		
33.044	-36.03	-5.18		37.9	-45.93	1.51		2.35
61.128	-60.9	-4.24		57.17	-56.61	-4.99		0.52
52.202	-13.73	-42.54		43.863	-9.75	-38.93		9.63
48.692	-73.31	23.72		48.242	-71.73	21.96		5.45
54.246	-1.21	-54.68		52.589	-3.71	-51.21		0.8
77.352	-78.74	-1.1		77.36	-79.27	-0.55		6.45
46.763	-65.42	21.06		47.32	-66.18	21.43		5.06
40.128	27.06	-66.03		43.427	17.4	-61.06		3.38
36.656	-15.87	-26.92		42.514	-6.95	-40.19		2.26
28.128	24.69	-54.14		29.84	19.42	-51.4		5.26
77.89	-121.98	64.85		78.533	-121.69	62.84		3.7
65.307	-96.89	45.34		64.824	-99.37	51.48		2.55
27.781	47.06	-70.96		26.265	51.11	-72.73		2.98
65.593	-102.17	46.34		65.047	-100.19	43.57		3.73
25.367	-20.48	-13.23		25.555	-22.14	-11.76		7.54
80.349	-126.78	74.68		80.271	-126.42	73.78		3.12
39.357	27.54	-65.04		38.697	33.19	-68.96		2.8
56.315	-34.45	-28.7		56.109	-33.73	-29.19		6.55
95.436	-67.09	-28.22		94.042	-67.72	-26.49		5.85
80.882	-56.82	-25.97		83.794	-55.56	-29.49		1.61
66.353	-48.14	-22.23		65.65	-46.54	-23.3		3.49
52.172	-39.85	-18.4		56.581	-46.77	-15.59		4.25
36.56	-30.26	-14.65		35.846	-27.65	-16.62		1.06
21.587	-20.85	-11.23		21.233	-21.51	-10.3		4.43
							Mean Color Difference	3.95
